# Differential reorganisation of cutaneous elastic fibres: a comparison of the *in vivo* effects of broadband ultraviolet B *versus* solar simulated radiation

**DOI:** 10.1039/c7pp00412e

**Published:** 2018-04-26

**Authors:** Nisamanee Charoenchon, Lesley E. Rhodes, Suzanne M. Pilkington, Mark D. Farrar, Rachel E. B. Watson

**Affiliations:** a Centre for Dermatology Research , Division of Musculoskeletal and Dermatological Sciences , School of Biological Sciences , Faculty of Biology , Medicine and Health , Manchester Academic Health Science Centre , The University of Manchester M13 9PT and The Dermatology Centre , Salford Royal NHS Foundation Trust , Salford M6 8HD , UK . Email: rachel.watson@manchester.ac.uk ; Tel: +44 (0)161 275 5505

## Abstract

Long-term exposure of human skin to ultraviolet radiation (UVR) in sunlight negatively impacts its appearance and function with photoaged skin having a characteristic leathery, rough appearance, with deep wrinkles. These clinical features of photodamage are thought to result from UVR-induced remodelling of the dermal extracellular matrix, particularly the elastic fibre system. There are few *in vivo* human data on the impact of acute UVR exposure on this fibre system and particularly solar-simulated radiation (SSR)-mediated effects. We examined the differential effect of broadband UVB and SSR on the human dermal elastic fibre system, and specifically the microfibrillar components fibrillin-1, fibulin-2 and fibulin-5. Healthy white Caucasian adults (skin type II–III) were recruited and irradiated with 3× their minimal erythema dose of broadband UVB (*n* = 6) or SSR (*n* = 6) on photoprotected buttock skin. Punch biopsies were taken 24 h after irradiation and from unirradiated control skin. Overall, histological assessment of elastic fibres revealed significantly less elastic fibre staining in broadband UVB (*P* = 0.004) or SSR (*P* = 0.04) irradiated skin compared to unirradiated control skin. Significantly less staining of fibrillin-1-positive microfibrils was also observed in the papillary dermis of UVB irradiated skin (*P* = 0.02) but not skin exposed to SSR. Conversely, immunohistochemistry for fibulin-5-positive microfibrils revealed significantly less expression in skin exposed to SSR (*P* = 0.04) but not to broadband UVB. There was no significant change in fibulin-2-positive microfibrils following either broadband UVB or SSR irradiation. Thus, broadband UVB and SSR mediate differential effects on individual components of the dermal elastic fibre network in human skin. Further human studies are required to explore the mechanisms underlying these findings and the impact of potential photoprotective agents.

## Abstract 2

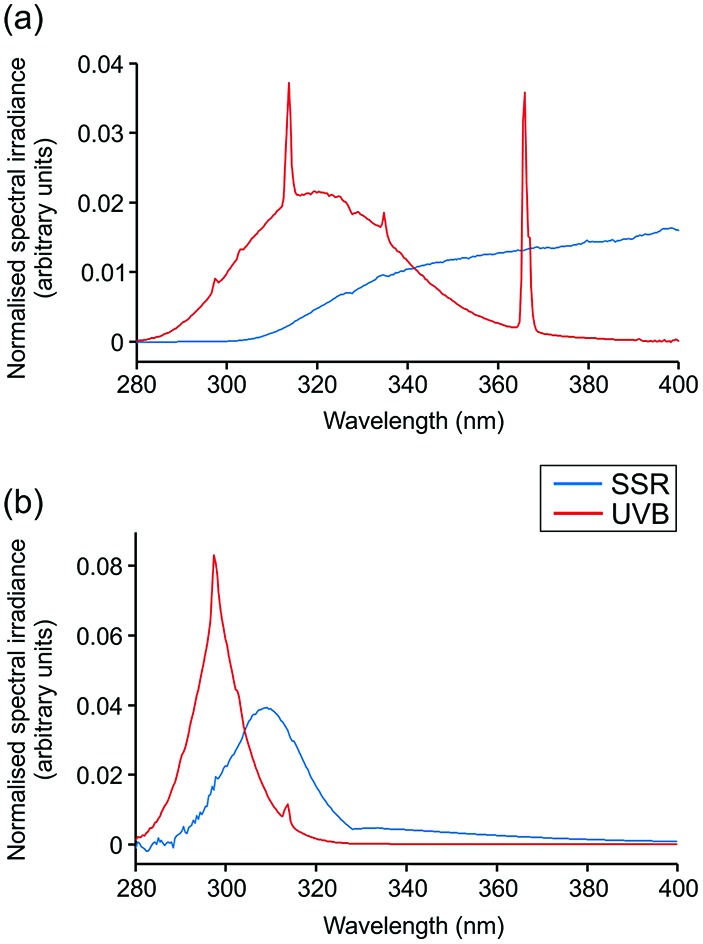
Long-term exposure of human skin to ultraviolet radiation (UVR) in sunlight negatively impacts its appearance and function.

## 1. Introduction

The skin protects the body from its external environment and provides a number of essential physiological roles such as thermoregulation and vitamin D synthesis. The structure of human skin is complex, comprising the superficial, continuously regenerating cell-rich epidermis and the deeper, relatively cell-poor dermis rich in extracellular matrix (ECM) proteins.^1^ Long-term exposure of skin to ultraviolet radiation (UVR) in sunlight causes photodamage and premature skin ageing.^1^–^4^ Clinically, photoaged skin presents as leathery and rough in appearance, with deep wrinkles and areas of mottled hyperpigmentation.^3^ These clinical changes in photoaged skin are thought to be driven by direct and indirect effects of chronic UVR exposure on the dermal ECM. Direct absorption of UVR can lead to structural changes and degradation of ECM proteins. Indirect effects may include generation of reactive oxygen species which subsequently damage ECM proteins, and induction of ECM-degrading enzymes such as matrix metalloproteinases (MMPs).^1^

The majority of ECM is produced by fibroblasts within the dermal compartment of skin and includes collagens, which provide strength, and elastic fibres which provide flexibility and recoil.^1^,^5^ Components of the elastic fibre system, particularly fibrillin-rich microfibrils, are especially susceptible to photodamage. We previously reported that these structures were lost from the papillary dermis of photoaged skin with only minimal clinical signs of photodamage.^6^ More recently, fibrillin-rich microfibril proteins (primarily fibrillin-1) were demonstrated to be enriched in amino acids which can directly absorb energy from UVR.^7^,^8^ Approximately 21% of the amino acids in the primary sequence of fibrillin-1 are UVR-chromophores which may account for the specific and early loss of fibrillin-rich microfibrils from photodamaged skin. Fibrillin-1 is the major family member of a group of modular proteins which also includes fibulin-2 and fibulin-5; these also localise to the dermal elastic fibre^9^ and were shown qualitatively to be susceptible to photodamage.^10^

There are very few data on the impact of acute UVR exposure on the elastic fibre network in human skin *in vivo*. An immunohistochemical study in two human volunteers reported loss of fibulin-2 and fibulin-5 following exposure of photoprotected buttock skin to a single UVB dose of twice their visual sunburn threshold (minimal erythema dose, MED), but the degree of loss was not quantified.^10^ In addition to the lack of *in vivo* human studies, mechanistic studies of photoageing, both *in vitro* and *in vivo*, have tended to focus on UVB-mediated effects, employing irradiation sources emitting broadband UVB. Solar simulated radiation (SSR) is more physiologically relevant, containing proportions of UVA and UVB similar to that of natural sunlight, but data on SSR-induced effects on parameters of photoageing are lacking. Thus, we aimed to assess the differential effects of acute exposure to broadband UVB and SSR on dermal ECM remodelling through histological and immunohistochemical staining, with digital image analysis and quantification of specific components of the elastic fibre system.

## 2. Materials and methods

### 2.1. Volunteers and study design

Healthy white Caucasian adults (*n* = 12; male and female, Fitzpatrick skin type II–III,^11^ aged 21–53 years) were recruited. Exclusion criteria were: history of skin cancer or photosensitivity; sunbathing/sunbed use in the previous 3 months; taking photoactive medication or supplements; pregnant or breast feeding. Volunteers had their MED of UVR determined then received a single standardised dose (3× MED) of either broadband UVB (*n* = 6) or SSR (*n* = 6) to photoprotected buttock skin. Samples were taken from buttock, a body site not usually exposed to solar irradiation; photoprotected buttock was therefore exposed to the specific dose of UVR from the designated light source or left unexposed as control for histological and immunohistochemical analysis of dermal matrix components. Ethical approval was granted by the Greater Manchester North Research Ethics Committee (refs: 11/NW/0567 and 08/H1006/79). Studies were conducted according to the Declaration of Helsinki, with participants providing written informed consent.

### 2.2. UVR exposures and skin sampling

**Fig. 1:**
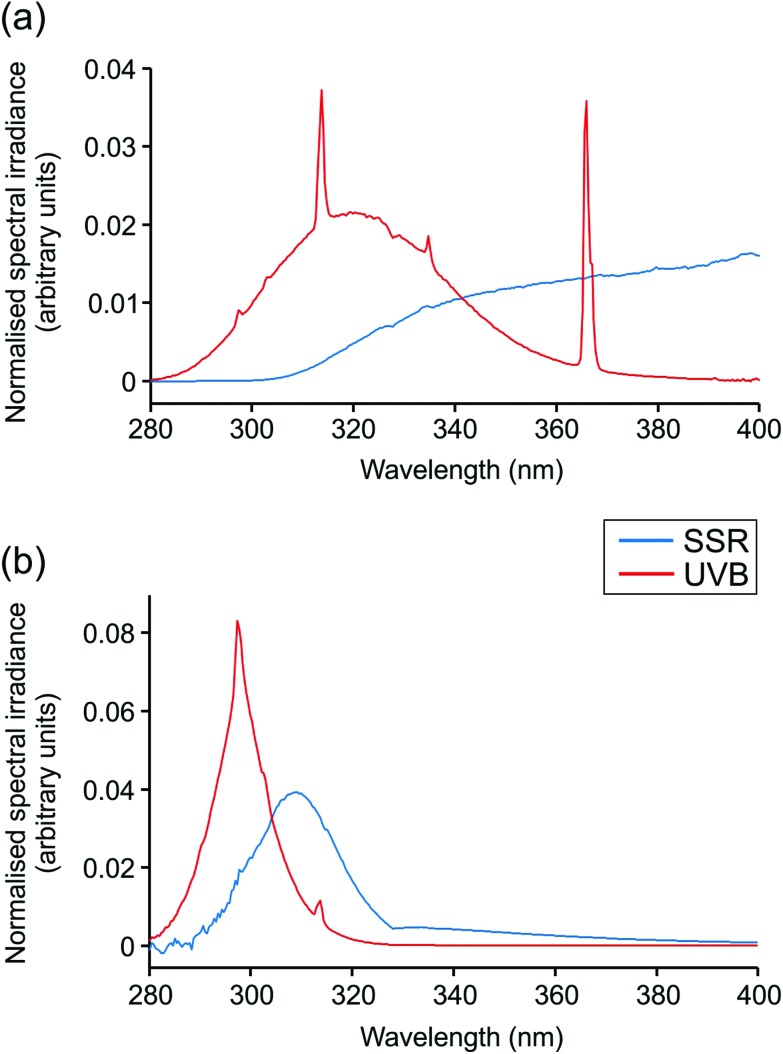
Spectral output of UVR sources. Spectral irradiance from 280–400 nm is shown for solar-simulated radiation (SSR) and broadband UVB sources used. (a) Total irradiance, (b) erythemally-weighted irradiance.

Irradiations were performed using a broadband UVB source (Waldmann UV6; 23% UVB, 77% UVA, range 280–400 nm; Herbert Waldmann GmbH, Villingen-Schwenningen, Germany) or solar simulator with UVR emission mimicking that of sunlight (5% UVB, 95% UVA, range 290–400 nm; Newport Spectra-Physics Ltd: Didcot, UK). Emission spectra are displayed in Fig. 1. The MED of all volunteers was assessed through application of a geometric dose series of UVR (erythemally weighted, 7–80 mJ cm^–2^) to photoprotected buttock skin. Twenty-four hours after irradiation, the MED was assessed visually and defined as the lowest dose producing just perceptible erythema. Samples of skin exposed to 3× MED broadband UVB or SSR and of unexposed skin were taken 24 h after irradiation by punch biopsy (5 mm) under local anaesthesia. Biopsies were embedded in OCT compound (Tissue-Tek® OCT; Sakura Finetek USA INC., Torrance, CA, USA), snap frozen in liquid nitrogen and stored at –80 °C prior to histological and immunohistochemical investigation.

### 2.3. Histology and immunohistochemistry

Frozen tissue was sectioned (7 μm) and stained to identify the entirety of the elastic fibre system using Weigert's resorcin fuchsin, or specific protein components of this system (fibrillin-1, fibulin-2, fibulin-5) by immunohistochemistry. All staining was performed in triplicate. In brief, to stain with Weigert's resorcin fuchsin, tissue was fixed in 4% (w/v) paraformaldehyde and incubated with Weigert's haematoxylin (Millipore; Darmstadt, Germany). Excess stain was removed with industrial methylated spirits (IMS) prior to immersion in Weigert's resorcin fuchsin for 90 min (Clin-Tech Limited; Guilford, UK). Stained sections were washed briefly in 70% IMS, dehydrated through serial alcohols, cleared in xylene and permanently mounted (DePex; Fisher Chemicals; Loughborough, UK). For immunohistochemistry, tissue was fixed in 4% (w/v) paraformaldehyde then permeabilised in 0.5% (v/v) Triton X-100 (Fisher Chemicals). Endogenous peroxidase activity was quenched by incubation in 0.6% (v/v) hydrogen peroxide in methanol, then non-specific antibody binding blocked by incubation with 3% (w/v) bovine serum albumin/1% (v/v) normal serum at room temperature for 1 h. Sections were incubated overnight at 4 °C with monoclonal anti-fibrillin-1 (clone 11C1.3, dilution 1 : 1000; NeoMarkers; Fremont, CA USA), polyclonal anti-fibulin-2 (HPA001934, dilution 1 : 1000; Atlas Antibodies AB; Stockholm, Sweden) or polyclonal anti-fibulin-5 (HPA000848, dilution 1 : 180; Atlas Antibodies AB; Stockholm, Sweden) and visualised using the Vector *Elite* ABC system (Vector Labs., Burlingame, CA, USA) according to manufacturer's instructions. Sections were dehydrated, cleared and mounted as described above.

### 2.4. Photomicrography, image analysis and statistics

All stained sections were randomised and blinded prior to image capture (Biozero-800 All-in-One microscope; Keyence; Osaka, Japan). For each parameter, nine sections were imaged and assessed. ImageJ (National Institutes of Health, Maryland, USA^12^) was used to quantify expression (positive staining) as percentage of the papillary dermal area from the dermal-epidermal junction (DEJ) to a depth of 100 μm across the whole section. The paired Student's *t*-test was used to assess differences in area stained between UVR-exposed and unexposed skin (SPSS v22, IBM Corp., New York, NY, USA) with significance accepted at *P* ≤ 0.05.

## 3. Results

### 3.1. Global effect of broadband UVB and SSR on the elastic fibre network

**Fig. 2:**
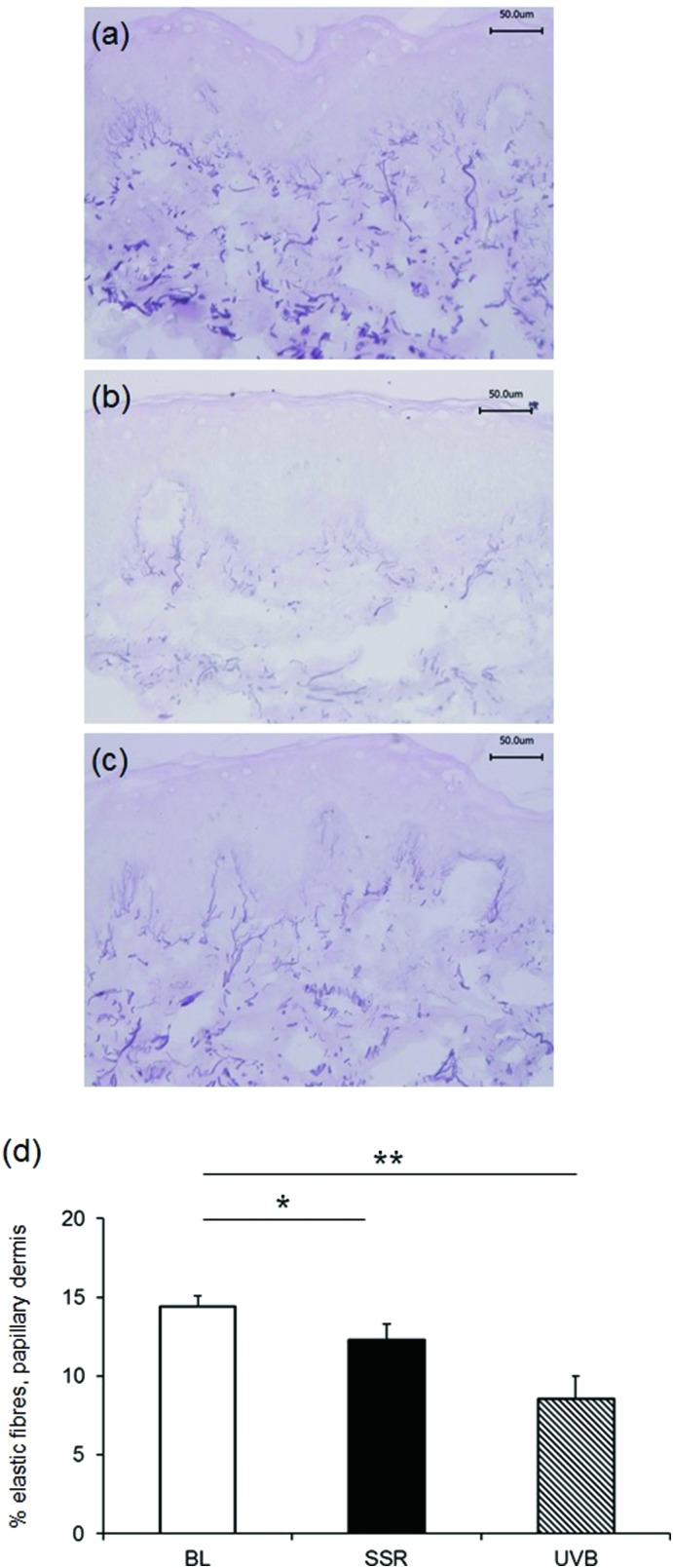
Papillary dermal elastic fibres are reduced following irradiation with broadband UVB or SSR. The distribution of elastic fibres was identified histologically using Weigert's resorcin fuchsin. Unirradiated photoprotected skin shows the classic arrangement of cutaneous elastic fibres (a). Irradiation with 3× MED broadband UVB (b) or SSR (c) resulted in significant breakdown and/or reorganisation of elastic fibres 24 h post-irradiation. Scale bar, 50 μm. (d) Image analysis was performed to quantify the area of the papillary dermis occupied by elastic fibres. **P < 0.01 and *P < 0.05 compared to unirradiated control.

Staining with Weigert's resorcin fuchsin to examine the global elastic fibre network in unirradiated control skin, revealed the characteristic candelabra architecture of fibrillin-rich oxytalan fibres close to the DEJ converging with perpendicular elaunin fibres in the papillary dermis that then coalesce with elastic fibres proper in the reticular and deeper dermis (Fig. 2a). Irradiation with a single 3× MED dose of broadband UVB (Fig. 2b) or SSR (Fig. 2c) to naïve, photoprotected skin significantly impacted on this pattern of distribution of elastic fibres in the papillary dermis. Image analysis and quantification showed that significantly less of the papillary dermis was positively stained in skin irradiated with broadband UVB (mean ± SEM 8.6 ± 1.4%) compared to unirradiated control skin (13.6 ± 0.7%; *P* = 0.004). A less marked difference was seen in skin irradiated with SSR (12.3 ± 1.0%; *P* = 0.04). A direct comparison of the effects on the elastic fibre components identified a significant difference between the different irradiation sources (UVB *versus* SSR; *P* = 0.05). Data is presented graphically in Fig. 2d.

### 3.2. Effect of broadband UVB and SSR on specific elastic fibre components

**Fig. 3:**
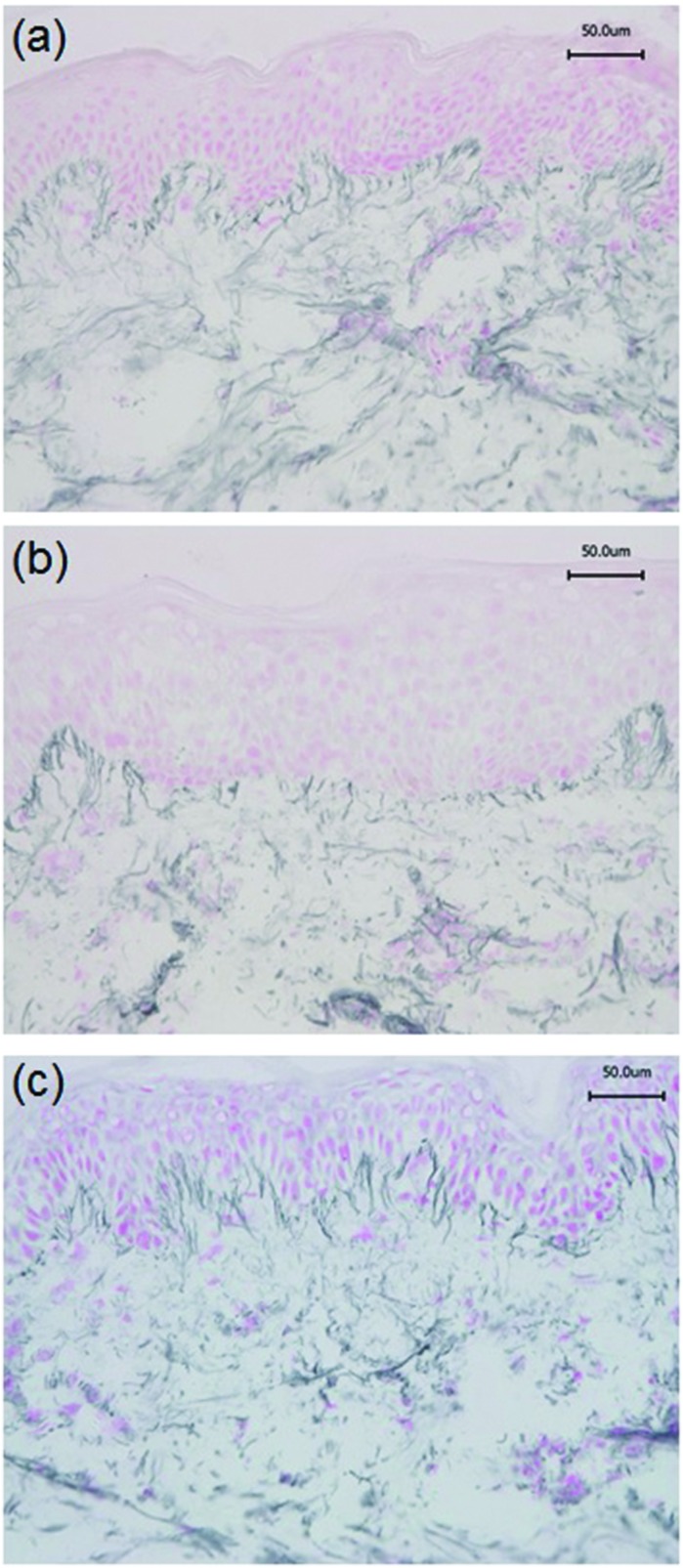
Broadband UVB but not SSR impacts on the arrangement and quantity of fibrillin-1 microfibrils in the papillary dermis. Immunohistochemistry was used to identify fibrillin-1-positive microfibrils in the papillary dermis; significant remodelling was observed following broadband UVB irradiation. (a) Unirradiated, (b) 3× MED broadband UVB, (c) 3× MED SSR. Scale bar, 50 μm.

**Fig. 4:**
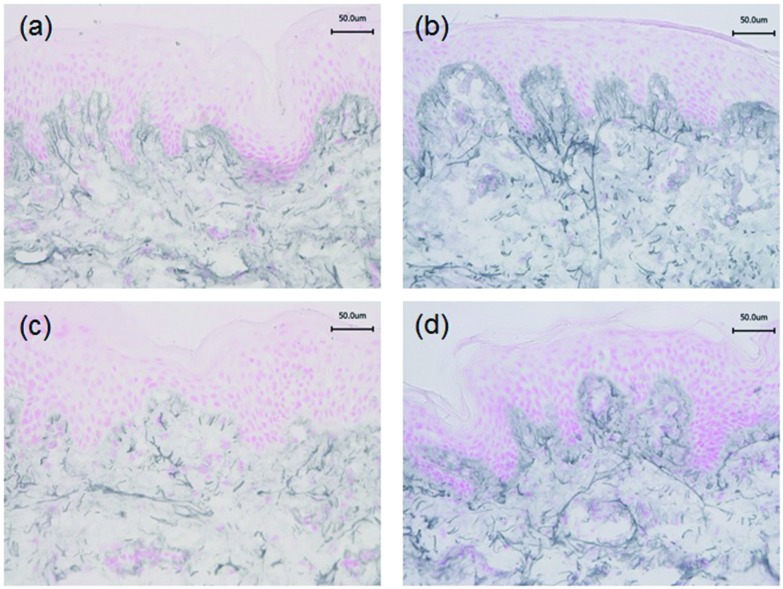
Neither broadband UVB nor SSR impacts on the arrangement and quantity of papillary dermal fibulin-2-positive microfibrils. Immunohistochemical staining and quantification of fibulin-2-positive microfibrils in the papillary dermis. No significant remodelling was seen following either irradiation protocol. (a), (b) Unirradiated, (c) 3× MED broadband UVB, (d) 3× MED SSR. Scale bar, 50 μm.

**Fig. 5:**
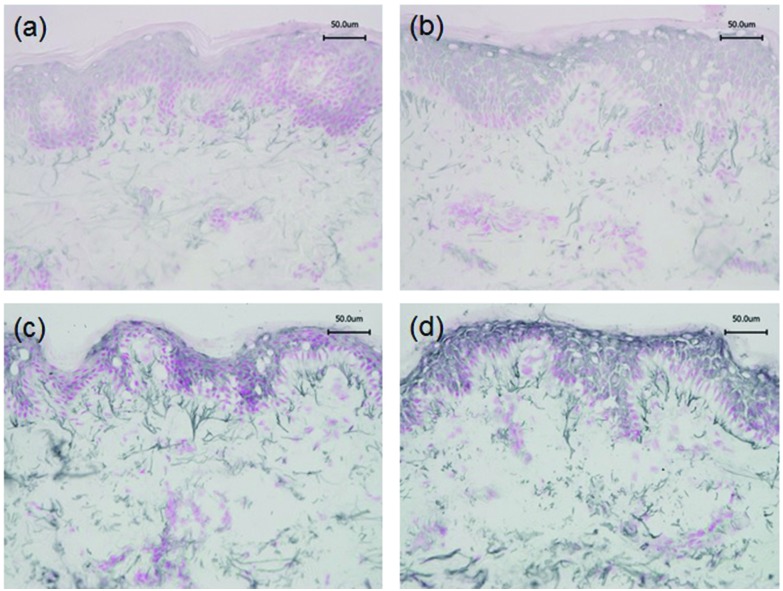
SSR but not broadband UVB impacts on the arrangement and quantity of papillary dermal fibulin-5-positive microfibrils. Immunohistochemical staining and quantification of fibulin-5-positive microfibrils in the papillary dermis. Significant remodelling was seen following irradiation with SSR. (a), (b) Unirradiated, (c) 3× MED broadband UVB, (d) 3× MED SSR. Scale bar, 50 μm.

**Fig. 6:**
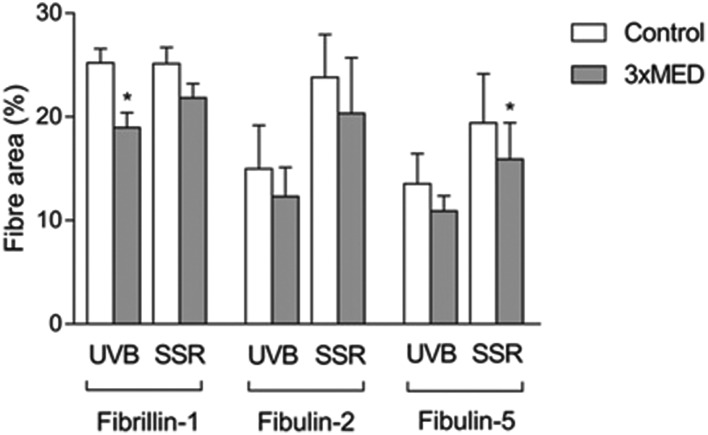
Quantification of immunohistochemical staining of elastic microfibril elements. Image analysis was used to quantify the coverage of elastic microfibrillar components in the papillary dermis. Data are mean ± SEM. *P ≤ 0.05 compared to respective controls.

Immunohistochemistry was performed to identify the distribution of elastic fibre proteins, namely fibrillin-1, fibulin-2 and fibulin-5-positive microfibrils. In baseline unirradiated skin, fibrillin-1-positive microfibrils (oxytalan fibres) were observed in the superficial papillary dermis, immediately below the DEJ covering 25.2 ± 1.5% of the papillary dermis (Fig. 3a). Irradiation with broadband UVB induced rearrangement and loss of these elastic fibre elements with significantly less papillary dermal expression (19.0 ± 1.5%; Fig. 3b) compared to unirradiated control skin (*P* = 0.02). Surprisingly, there was little alteration in the distribution or amount of fibrillin-1-positive microfibrils in SSR-irradiated skin, with 21.9 ± 1.4% of the papillary dermis occupied by positively stained fibres (*P* = 0.11 compared to unirradiated control skin; Fig. 3c). Fibulin-2-positive microfibrils also appeared to emanate from the DEJ, but were generally of shorter length than fibrillin-1-positive microfibrils, occupying less of the papillary dermis, with greater inter-volunteer variability. Irradiation with either source of UVR did not elicit significant remodelling of fibulin-2 with irradiated and control skin demonstrating similar levels of expression (12.3 ± 1.2% and 15.0 ± 1.7% respectively for broadband UVB (*P* = 0.10) and 20.3 ± 2.2% and 23.8 ± 1.7% respectively for SSR (*P* = 0.06; Fig. 4)). Fibulin-5-positive microfibrils had a similar distribution to fibrillin-1-positive microfibrils with longer fibres coalescing with elaunin fibres in the papillary dermis but with similar variability in content as observed with fibulin-2 (Fig. 5a and b). No significant difference was observed between skin irradiated with broadband UVB (Fig. 5c) and the unirradiated control (10.9 ± 0.6% and 13.5 ± 1.2% respectively, *P* = 0.11). However, irradiation with SSR significantly reduced the expression of fibulin-5 microfibrillar elements in the papillary dermis (15.9 ± 1.4%; Fig. 5d) compared to unirradiated control skin (19.4 ± 1.9%, *P* = 0.04). Quantification of image analysis for fibulins-2 and -5 is presented graphically in Fig. 6.

## 4. Discussion

Photoageing as a result of long-term exposure to solar UVR is characterised by skin wrinkling, uneven pigmentation and reduced resilience.^3^ Histologically this presents as elastosis, loss of fibrillin-rich microfibrils, and altered patterns of fibulin deposition.^1^,^6^,^12^–^15^ There are few *in vivo* studies on the impact of acute UVR exposure on the human dermal ECM and many have focussed on effects mediated by UVB which, despite its higher energy, only comprises approximately 5% of the total UVR in sunlight. In order to better understand sunlight-mediated damage of the dermal ECM in healthy humans, we have performed a comparative study of broadband UVB and SSR examining global changes in the elastic fibre network and effects on the specific elastic fibre components fibrillin-1, fibulin-2 and fibulin-5. We found that in naïve, photoprotected buttock skin (*i.e.* skin which is rarely, if ever, is exposed to solar UVR) a single 3× MED dose of broadband UVB or SSR impacts the organisation and abundance of the elastic fibre network as a whole, with significantly reduced papillary dermal staining as compared to unirradiated control skin, the effect being greater in UVB-irradiated skin. Assessment of individual fibre components showed reduction in expression of fibrillin-1 specifically following UVB exposure and fibulin-5 following SSR exposure; it is possible that, as SSR contains a higher proportion of UVA than that emitted by the broadband UVB source, there is a differential effect due to either the primary structure of the assembly or the location of the molecule on the oxytalan fibre.

Our findings build on the qualitative study of Kadoya *et al.*^10^ who reported that fibulin-2- and fibulin-5-containing microfibrils were visibly reduced on immunohistochemical staining 2 days after a single exposure of 2× MED UVB, with the reduction in fibulin-5 being more marked. However, the UVB-induced effect on fibrillin-1 was not examined and details of the UVB source were not given. We similarly found a differential effect on fibulin-2 and fibulin-5 following irradiation with SSR, but not with broadband UVB, potentially attributable to differences in penetration depth^16^ and/or mechanisms of fibre degradation and remodelling.

The mechanisms by which elastic fibre remodelling occurs in human skin require further investigation. We previously reported that most elastic fibre-associated proteins contain a high proportion (up to 21%) of UVR-absorbing amino acids, namely Cys, His, Phe, Trp and Tyr, which may predispose them to direct photodegradation.^7^,^8^ This was supported by *in vitro* demonstration that these chromophore-rich ECM components are selectively degraded by low dose UVR, with low-chromophore content proteins (*e.g.* type I collagen) relatively unaffected. Indirect mechanisms may also contribute to UVR-induced elastic fibre remodelling. Exposure to UVR generates reactive oxygen species that can react with and damage DNA, lipids and proteins.^17^,^18^ Photo-oxidation of proteins can result in structural changes, aggregation and/or fragmentation.^19^ Furthermore, UVR induces upregulation of MMP release and activation and the infiltration of immune cells into the dermis which release other enzymes (*e.g.* neutrophil elastase), all of which may potentially contribute to degradation and remodelling of resident elastic fibres.^20^–^22^ The differential effects of broadband UVB and SSR on these potential mechanisms and relation to changes in the elastic fibre network *in vivo* require further investigation.

Chronic photodamage is the product of many years of sunlight exposure, which incrementally alters the elastic fibre system of the skin. Personal levels of sunlight exposure are not constant; variables such as latitude, season, and sun exposure/protection behaviour all contribute.^23^–^25^ The impact of ethnicity and skin pigmentation on dermal structure and responses to UVR exposure must also be considered.^26^ Thus, to more accurately model chronic photodamage in human skin, studies employing repeated low dose (sub-erythemal) SSR exposures in different skin types are required. This approach may provide better understanding of the pathways which underpin the pathology of photoageing and assist in the development of human experimental systems for the testing of potential photoprotective agents.

## Conflicts of interest

There are no conflicts of interest to declare.
